# Model informed dose regimen optimizing in development of leritrelvir for the treatment of mild or moderate COVID-19

**DOI:** 10.3389/fphar.2024.1449583

**Published:** 2024-08-28

**Authors:** Kun Wang, Haijun Li, Youyun Li, Fengyan Xu, Zhongyi Sun, Yuting Yang, Jufang Huang, Xiaoxin Chen

**Affiliations:** ^1^ Shanghai Qiangshi Information Technology Co., Ltd., Shanghai, China; ^2^ Guangdong Raynovent Biotech Co., Ltd., Guangzhou, China; ^3^ Department of Anatomy and Neurobiology, School of Basic Medical Science, Central South University, Changsha, China

**Keywords:** leritrelvir, RAY1216, 3CL protease, COVID-19, pharmacokinctics

## Abstract

**Introduction:**

Leritrelvir (RAY1216) acts as a main protease inhibitor that hinders the cleavage of viral precursor proteins, thereby inhibiting virus replication of SARS-CoV-2). This antiviral mechanism has shown significant efficacy against the novel coronavirus. Preclinical studies have demonstrated the potent antiviral activity and favorable safety profile of this compound. This study aims to develop a pharmacokinetic model for leritrelvir, with and without ritonavir as a pharmacokinetic enhancer and to evaluate the necessity of co-administration with ritonavir and to investigate different dosage regimens.

**Method:**

The model establishment was based on plasma concentration data from a phase I trial involving 72 subjects in single-ascending dose (SAD), multiple-ascending dose (MAD), and a food effect cohort. Analysis was conducted using a nonlinear mixed-effects model, and clinical trial simulations were carried out.

**Results:**

The findings of this study demonstrate a favorable safety profile for leritrelvir. With simulation suggests that a 400 mg thrice-daily (TID) regimen may be optimal to maintain the trough concentrations (C_trough_) above levels required for inhibiiting viral replication. While ritonavir was found to enhance exposure, it was deemed unnecessary. Gender and food consumption were identified as significant covariates affecting pharmacokinetic parameters, however, no dose adjustments were deemed necessary.

**Discussion:**

This findings supported by subsequent phase II and phase III trials validated the appropriateness of a 400 mg TID regimen for the administration of leritrelvir.

## 1 Introduction

The emergence of the novel coronavirus, leading to the global pandemic of Corona Virus Disease 2019 (COVID-19), has posed a significant threat to global public health security (Muralidar et al., 2020). Despite the development and distribution of vaccines and neutralizing therapeutic antibodies since 2021, the rapid evolution of virus variants has resulted in reduced vaccine efficacy and breakthrough infections. There is a pressing need for orally administered antiviral medications that exhibit potent antiviral activity, specificity, efficacy against multiple mutant strains, and ease of transportation and distribution for early use in infections.

The 3CL protease (3CLpro) plays a crucial role in the replication process of coronaviruses (Zhang et al., 2023), highly conserved in coronavirus sequences with low off-target risks. Its amino acid sequence in the activation center has low homology with human proteases, reducing safety concerns for human use (Ho et al., 2023). Compared to the spike protein, 3CLpro exhibits a lower likelihood of mutation, rendering it a promising target for drug development. As a SARS-CoV-2 3CLpro inhibitor, nirmatrelvir-ritonavir (Paxlovid) received emergency authorization in the United States on 22 December 2021, for the treatment of non-hospitalized, mild to moderate COVID-19 patients aged 12 and above at high risk of developing severe illness (Iketani et al., 2023). In November 2022, ensitrelvir, another 3CLpro inhibitor, was approved in Japan (Ip et al., 2023). Following the lifting of a 2-year lockdown in China in 2022, the nation faced widespread COVID-19 outbreaks. On 11 February 2022, China’s National Medical Products Administration conducted an emergency review and approval through a specialized drug approval process, conditionally approving the import registration of Paxlovid (China grants conditional approval for, 2022). Nevertheless, the supply of Paxlovid fell short of meeting the substantial demands of the Chinese market. Furthermore, the co-administration of Paxlovid including ritonavir presents limitations in clinical application for patients with underlying conditions, such as cardiovascular disease, diabetes, and tumors. Thereby affecting a significant portion of the population in China, particularly the elderly.

In light of the pressing clinical need arising from the persistent COVID-19 pandemic, Guangdong Raynovent Biotech Co., Ltd. has developed the 3CLpro inhibitor leritrelvir (RAY1216) leveraging their expertise in protease inhibitors. This compound specifically targets the main protease of SARS-CoV-2, preventing the cleavage of viral precursor proteins, and consequently impeding viral replication, thereby demonstrating antiviral effectiveness against the novel coronavirus. Preclinical investigations have demonstrated that leritrelvir’s potent antiviral properties, favorable pharmacokinetics, and a high safety profile, suggesting its potential utility in clinical interventions for COVID-19. *In vitro* pharmacological analyses have revealed that leritrelvir displays notable inhibitory activity against the SARS-CoV-2 βCoV/KOR/KCDC03/2020 strain in Vero cells, with an half maximal inhibitory concentration (IC_50_) of 36 nM and 90% inhibitory concentration (IC_90_) of 92 nM (Chen et al., 2023). The compound demonstrates notable inhibitory efficacy against multiple viral strains, as evidenced by concentration for 90% of maximal effect (EC_90_) values of 228 nM, 351 nM, 688 nM, 254 nM, 208 nM, 363 nM and 415 nMfor the wild-type strain, Alpha variant, Beta variant, Delta variant, Omicron BA.1, Omicron BA.5 and Omicron XBB.1.9.1 variant, respectively (Chen et al., 2024). Similar to those observed for the control compound Nirmatrelvir (PF-07321332). In an *in vivo* efficacy study utilizing K18-hACE2 mice infected with the Delta variant, leritrelvir demonstrated inhibition effects at doses of 300 mg/kg and 600 mg/kg, with inhibition rates of 42.86% and 100%, respectively, indicating a dose-dependent response (Chen et al., 2023). Furthermore, leritrelvir significantly reduced viral titers in the lungs of mice after infection and exhibited favorable enhancements in lung pathology. CYP3A serves as the primary metabolizing enzyme for leritrelvir, with minimal to no involvement from other isoforms such as CYP1A2, CYP2B6, CYP2C8, CYP2C9, CYP2C19, and CYP2D6 (in-house data). The population pharmacokinetics (PopPK) characteristics of leritrelvir in humans are currently unknown as it is undergoing its first human study. However, based on non-clinical toxicology studies, leritrelvir demonstrates a large safety margin. In order to ensure the safety of subjects, the initial dose of leritrelvir for single-drug administration in this study is set at 400 mg, while the initial dose is reduced to 200 mg when co-administered with ritonavir. This analysis aimed to characterize the pharmacokinetics (PK) of leritrelvir when administered alone and in combination with ritonavir (RTV), investigate the influence of various intrinsic and extrinsic factors on the PK of leritrelvir, and provide insights for decision-making in the development of adjunctive medications. This study utilized population pharmacokinetic analysis as a crucial tool in model-informed drug development to address questions regarding the necessity of co-administration with RTV, the potential use of leritrelvir as a standalone medication, appropriate dosage for future clinical applications, and the suitability of twice-daily (BID) or thrice-daily (TID) dosing.

## 2 Methods

### 2.1 Study design

**FIGURE 1:**
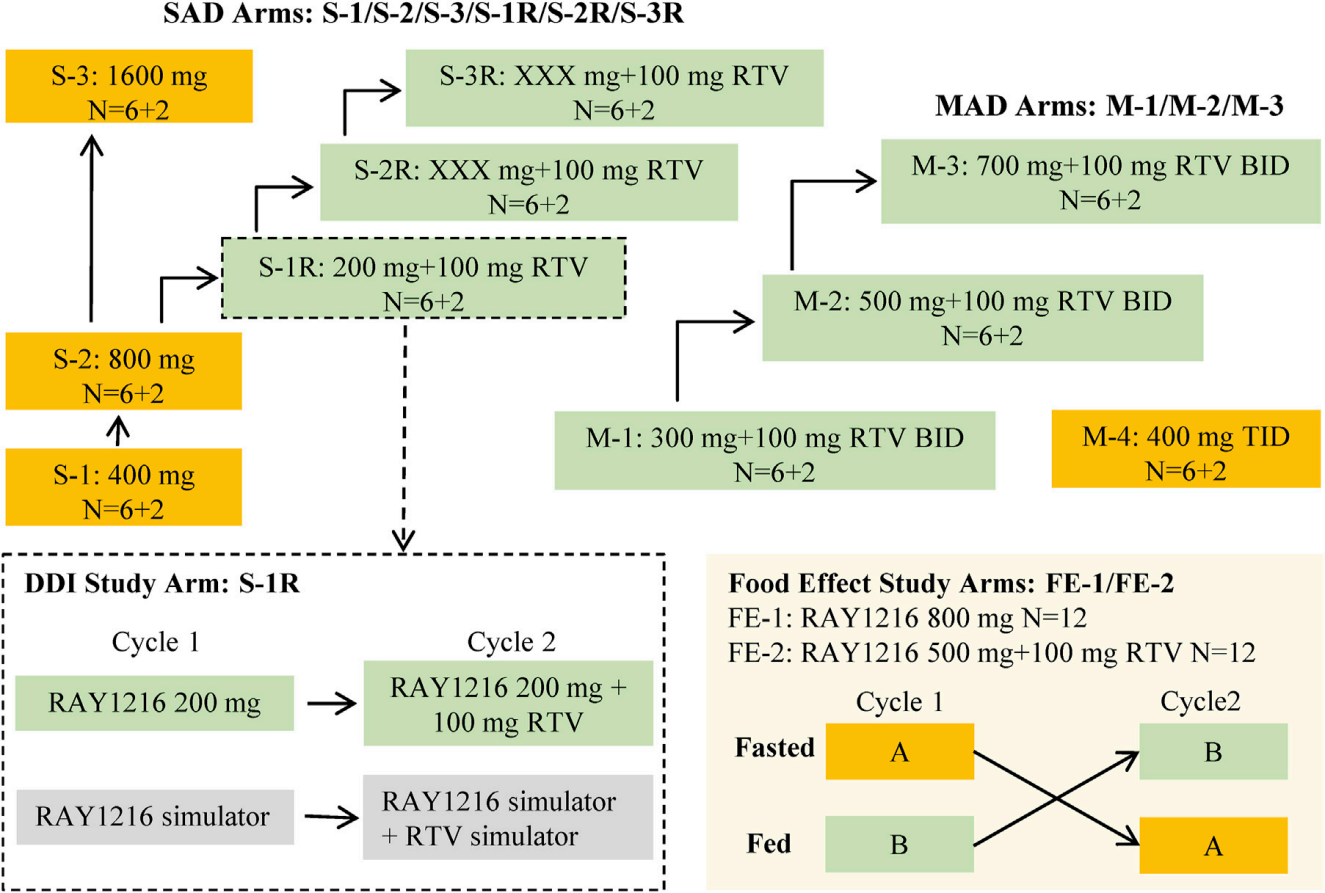
Overall study design diagram. The study comprised three distinct components: a single-ascending dose study (SAD), a food effect study, and a multiple-ascending dose study (MAD). The SAD portion of the study was divided into two parts: Part 1 involved a dose escalation study of single-agent administration of RAY1216 tablets (S-1, S-2, and S-3), while Part 2 focused on the combination of RAY1216 tablets with 100 mg RTV (S-1R, S-2R, and S-3R). It should be noted that the S-2R and S-3R groups were initially planned but were ultimately not conducted due to dose-undetermined factors. The food effect study (FE) entails the administration of single doses of RAY1216 tablets at 800 mg (FE-1) and in combination with RTV at 500 mg (FE-2) to groups of 12 subjects. Group A undergoes a fasting administration cycle followed by a postprandial administration cycle, while Group B experiences the reverse order of administration cycles. The two cycles consist of crossover administration with a washout period lasting a minimum of 8 days. The MAD involves the co-administration of RAY1216 tablets (300 mg, 500 mg, and 700 mg) with 100 mg RTV in a BID dosing regimen, with escalating doses administered over 5 consecutive days and solely in the morning on the 5th day. Furthermore, a 400 mg RAY1216 single-agent TID dosing regimen administered over 5 consecutive days with administration limited to the morning on the 5th day. In the diagram, “6 + 2” indicates that each group comprises 8 enrolled subjects, with 6 subjects receiving the investigational drug and 2 subjects receiving a placebo.

The data obtained from a phase I clinical trial assessing the safety, tolerability, pharmacokinetics, and impact of food on leritrelvir Pharmacokinetics in healthy adult subjects (NCT05829551) were utilized in the PopPK analysis. The trial was designed as a randomized, double-blind, placebo-controlled study involving escalating single and multiple doses. The study design is outlined in Figure 1. In the single-ascending dose (SAD) arm, participants received leritrelvir tablets alone or with 100 mg of RTV. In the multiple-ascending dose (MAD) arm, leritrelvir tablets were given alone or with 100 mg RTV twice-daily on Days 1–4, followed by a single dose on Day 5, or thrice-daily on Days 1–4, followed by a single dose on Day 5. In the food consumption study arm, leritrelvir tablets alone or with 100 mg RTV were administered once during each dosing period after an overnight fast of at least 10 h or following a standard meal (high-fat, high-calorie) on Day 1. Each dosing period included a single administration, totaling two administrations, with a minimum interval of 8 days between periods.

### 2.2 PopPK analysis

Leritrelvir PK data was analyzed utilizing nonlinear mixed-effects modeling (NONMEM) (Lindstrom and Bates, 1990) and implemented with NONMEM version 7.5 (ICON Development Solutions. Ellicott City, Maryland, USA). The estimation algorithm used was the first-order conditional estimation method with interaction (FOCEI) of interindividual variability and residual variability. Perl Speaks NONMEM (PsN) version 4.2 (Uppsala University, Sweden) (Lindbom et al., 2004; Lindbom et al., 2005) and R version 4.1.2 were used as tools for executing NONMEM and summarizing outcomes. The model evaluated the impact of age, weight, body mass index, gender, albumin, alanine aminotransferase, aspartate aminotransferase, alkaline phosphatase, serum creatinine, creatinine clearance rate, total bilirubin, gamma-glutamyl transpeptidase, food consumption, and concomitant medication on the pharmacokinetics of leritrelvir. The final PopPK model was determined through a stepwise forward addition (*p* < 0.01) and backward deletion (*p* < 0.001) approach. The specifics of the model development process are outlined in the Supplementary Material. Evaluation of the final PopPK model was conducted using goodness-of-fit plots, prediction-corrected visual predictive check (pcVPC) (Bergstrand et al., 2011), bootstrapping (Ette, 1997), and shrinkage assessments (Savic and Karlsson, 2009).

### 2.3 PopPK model simulations

#### 2.3.1 Target concentration

To determine the optimal dosing regimen for achieving the desired target, the final population pk model was utilized to simulate concentration-time profiles under various conditions. Trough concentration (C_trough_) within 1 day after the first dose on both the 1st and 5th days was calculated. Specifically, concentrations were assessed at 6 h, 12 h, and 24 h when the drug was given three times a day (once at 8:00, 14:00, and 20:00 each day), and at 12 h and 24 h on the 1^st^ day when given twice a day (once at 8:00 and 20:00 each day). Additionally, concentration was measured at 12 h on the 5th day when administered twice a day. In the process of model development, we also considered inter-occasion variability for multiple dosing data, such as inter-occasion variability in clearance (CL) and volume (V_c_). After multiple dosing, the suitability of different time points (12 h, 24 h, 48 h, 72 h, 84 h, 96 h, and 108 h after first dose) set as the boundaries for inter-occasion variability was evaluated using the objective function and goodness-of-fit plots. Since eating food can increase the pharmacokinetic exposure and without safety issues, the study simulated two scenarios under the fasted state: 1. Single use of leritrelvir was simulated in 1000 subjects, comprising male and female patients in a fasting state (n = 500 each). 2. Co-administration of leritrelvir and RTV was simulated in 1000 subjects, with an equal distribution of male and female patients in a fasting state (n = 500 each).

Concentrations of leritrelvir needed to achieve EC_90_ against the Omicron variant and the wild strain of COVID-19 were found to be 209.7 ng/mL and 165 ng/mL, respectively. The percentages of subjects with trough concentrations surpassing 1, 3, and 5 times the EC_90_ on the 1st and 5th days were calculated. Two simulation scenarios were considered: Scenario 1 involved a single use of leritrelvir, with doses of 300, 400, 500, and 600 mg given TID at 8:00, 14:00, and 20:00 daily for five consecutive days. Scenario 2 included the co-administration of leritrelvir and RTV, with doses of 200 and 300 mg given BID at 8:00 and 20:00 daily for five consecutive days.

#### 2.3.2 Impact of gender and food on exposure

In the PK analysis population, gender and food were identified as significant covariates. To further evaluate the impact of gender and food on leritrelvir, concentration-time profiles were generated through deterministic simulation using the individual PK parameters estimated from the final PopPK model.

## 3 Results

### 3.1 PopPK analysis dataset

The demographic characteristics of the study participants are outlined in Table 1. A total of 1571 leritrelvir concentrations from 72 subjects were included in the PK analysis dataset. Data points below the lower limit of quantification, representing 8.72% (150/1721) of the dataset, were excluded from the analysis. The leritrelvir plasma concentrations over time, stratified by dose arm, are depicted in Supplementary Figure S1.

**TABLE 1:** Baseline population characteristics in the analysis datasets.

Item (unit) N	All subjects (N = 72)
PK samples	1571
Continuous Variable (median [min, max])	
Age (AGE, years)	35.0 (19.0, 48.0)
Body weight (WT, kg)	60.6 (48.2, 90.8)
Height (HT, cm)	163 (148, 191)
Body mass index (BMI, kg/m2)^a^	22.9 (18.4, 27.9)
Albumin (ALB, g/L)	43.9 (39.5, 52.4)
Aspartate aminotransferase (AST, U/L)	18.8 (11.8, 31.7)
Alanine aminotransferase (ALT, U/L)	13.8 (7.00, 42.9)
Alkaline phosphatase (ALP, U/L)	66.2 (43.0, 137)
γ- Glutamine transferase (GGT, U/L)	18.4 (9.00, 104)
Total bilirubin (BILI, μmol/L)	13.2 (6.50, 23.8)
Creatinine (CREAT, μmol/L)	63.8 (37.3, 93.8)
Creatinine clearance (CRCL, mL/min)^b^	115 (75.6, 170)
Categorical Variable (category, N)	
Sex	
Male	36 (50.00%)
Female	36 (50.00%)
Food consumption^c^	
Fasted	78 (76.47%)
Fed	24 (23.53%)
Drug combination^c^	
Mono	54 (52.94%)
Comb	48 (47.06%)

^a^ BMI, is calculated as follows: Body weight [kg/Height (m^2^).

^b^ Creatinine clearance is calculated as follows: (140 – Age [year]) × Body weight [kg]/(72 × Creatinine [μmol/L]/88.4) × 0.85 (if Female).

Abbreviations: PK, pharmacokinetics; min, Minimum; max, Maximum; N, number of subjects.

^c^ The N = 102 for food consumption and drug combination. Due to 30 subjects in the food consumption study with the crossover design and a washout period, each subject was assigned to two IDs, in the NONMEM, model.

### 3.2 PopPK analysis

#### 3.2.1 Final PopPK results

The pharmacokinetics of leritrelvir following oral administration were well described by a 2-compartment linear disposition model with first-order elimination. The PopPK model was parameterized in terms of central compartment clearance (CL), central compartment volume (V_c_), absorption rate constant (K_a_), inter-compartmental clearance (Q), peripheral compartment volume (V_p_), zero-order absorption duration (D_1_), absorption lag time (ALAG_1_), and relative bioavailability (F_1_). The relative bioavailability was set at a fixed value of 1 with other parameters being estimated separately for leritrelvir alone and in combination with RTV. Gender was identified as a significant covariate affecting clearance, while food consumption was found to significantly impact bioavailability, absorption rate constant, and zero-order absorption duration.

Considering preclinical data and the drug’s metabolism through the CYP3A4 pathway, potential autoinhibition or induction effects were taken into consideration. Therefore, the modeling process investigated the inter-occasion variability in CL and V_c_ parameters at 12 h, 24 h, 48 h, 72 h, 84 h, 96 h, and 108 h post-multiple dosing, as well as changes in pharmacokinetic parameters. The results indicated that the inter-occasion variability in CL and V_c_ at 12 h and 96 h post-multiple dosing significantly improved the model’s goodness of fit. This suggests that leritrelvir exhibits auto-inhibition after multiple dosing at 12 h and auto-induction at 96 h, thereby improving the model’s description of the observed data. This model serves as the final base model. The study explored the incorporation of proportional errors near the time of dosing to account for the increased variability in drug absorption observed during the initial period following administration compared to later periods. Analysis of different time points (0.5 h, 1 h, 2 h, 3 h, 4 h, and 5 h) as cutoff points indicated that including proportional errors within the first 0–2 h post-dosing in fed subjects enhanced the accuracy of the absorption phase. The final PopPK model parameters for leritrelvir were estimated and are detailed in Table 2.

**TABLE 2:** PopPK parameters of Leritrelvir and bootstrap results.

Parameter	Population model estimates (%RSE) [95% CI]		Bootstrapping
			Median (95% CI)
Fixed effect parameters			
Clearance, CL (L/h)	Mono	44.4 (6.68) [39–50.6]	44.2 (39.0–50.7)
	Comb	12.2 (8.02) [10.4–14.3]	12.2 (10.7–14.0)
Volume distribution of peripheral compartment, V_c_ (L)	Mono	18.9 (18) [13.3–26.8]	18.7 (14.3–25.3)
	Comb	9.78 (27.1) [5.75–16.7]	9.90 (7.68–13.2)
Absorption rate, K_a_ (hr^-1^)	Mono	0.293 (6.95) [0.256–0.336]	0.292 (0.258–0.329)
	Comb	0.169 (7.83) [0.145–0.197]	0.170 (0.154–0.185)
Intercompartment clearance between the central compartment and peripheral compartment, Q (L/h)	Mono	5.57 (6.47) [4.91–6.32]	5.49 (3.18–8.02)
	Comb	0.611 (15.8) [0.449–0.833]	0.609 (0.441–0.9)
Volume distribution of peripheral compartment, V_p_ (L)	Mono	58.2 (7.49) [50.3–67.4]	58.2 (44.7–79.5)
	Comb	9.30 (11.5) [7.42–11.7]	9.24 (6.93–12.8)
Duration of zero order absorption, D_1_ (hr)	Mono	0.0722 (38) [0.0343–0.152]	0.078 (0.0359–0.171)
	Comb	0.0684 (49.8) [0.0258–0.182]	0.0719 (0.0412–0.129)
Absorption lag time, ALAG_1_ (hr)	Mono	0.189 (2.33) [0.18–0.197]	0.188 (0.164–0.2)
	Comb	0.166 (3.22) [0.156–0.177]	0.166 (0.131–0.185)
Relative bioavailability,F_1_			1, FIX
Impact of food consumption on F_1_, F_1_.FED		1.51 (5.9) [1.34–1.69]	1.50 (1.25–1.82)
Impact of food consumption on K_a_, K_a_.FED		23.2 (113) [2.51–214]	21.3 (10.8–41)
Impact of food consumption on D_1_, D_1_.FED		1.34 (12.6) [1.05–1.71]	1.33 (1.22–1.49)
Autoinhibition effect on CL after multiple dose for 12 h, CL.OC1.INH		0.626 (7.84) [0.537–0.73]	0.626 (0.543–0.71)
Autoinduction effect on CL after multiple dose for 96 h, CL.OC2.IND		1.51 (6.18) [1.34–1.7]	1.51 (1.35–1.68)
Impact of gender on CL, CL.sex		0.800 (7.62) [0.689–0.929]	0.802 (0.708–0.91)
Inter-individual variability (%)			
CL		30.6 (10.3) [23.6–36.2]	29.9 (25.5–34.6)
V_c_		80.8 (12.6) [57.6–98.7]	79.8 (64.7–93.4)
K_a_		26.1 (14) [17.5–32.4]	25.3 (20.7–29.4)
V_p_		44.7 (12) [32.6–54.2]	43.6 (31–52.5)
D_1_		155 (13.6) [106–192]	152 (114–189)
Occasion variability of CL after multiple dose for 12 h, OC1.CL		19.3 (21.9) [7.22–26.3]	18.7 (10–29.3)
Occasion variability of CL after multiple dose for 96 h, OC2.CL			
Occasion variability of V_c_ after multiple dose for 12 h, OC1.V_c_		54.4 (19.2) [27–72.1]	53.1 (34–82.5)
Occasion variability of V_c_ after multiple dose for 96 h, OC2.V_c_			
Residual variability			
Proportional residual (%)		24.4 (2.06) [23.4–25.4]	24.2 (22.4–26.1)
Proportional error of fed subjects within 2 h after dose (%)		70.2 (16.8) [41.1–90.3]	70.5 (56.4–80.7)

OC1 refers to occasions where multiple doses are administered with a dosing interval of ≥12 h, while OC2 refers to occasions where multiple doses are administered with a dosing interval of ≥96 h. SEX represents gender, and FED represents feeding status. $\eta_{CL,i},\eta_{Vc,i}$ , $\eta_{Ka,i}$ , $\eta_{Vp,i}$ , and $\eta_{D1,i}$ denote interindividual variability for the ith subject’s CL, V_c_, K_a_, V_p_, and D_1_, respectively. $\eta_{OC1,i}\;and\;\eta_{OC2,i}$ represent interindividual variability for OC1 and OC2; CI, confidence interval; IIV, interindividual variability; RSE, relative standard error; The shrinkages were 3.12%, 13.3%, 11.5%, 11.1%, and 18.8%, for CL, V_c_, K_a_, V_p_, and D_1_, respectively.

When leritrelvir is used alone, the pharmacokinetic parameters of leritrelvir are:

$${CL}_{i}\left(L/hr\right)=44.4\times \exp\left(-0.469\times \text{OC}1+0.412\times \text{OC}2-0.224\times \text{SEX}+OC1\times \eta_{OC1,i}+{OC2\times \eta }_{OC2,i}+\eta_{CL,i}\right)$$

$$V_{c,i}\left(L\right)=18.9\times \exp\left(OC1\times \eta_{OC1,i}+{OC2\times \eta }_{OC2,i}+\eta_{Vc,i}\right)$$

$$K_{a,i}\left(1/\text{hr}\right)=0.293\times \exp\left(0.291\times \text{FED}+\eta_{Ka,i}\right)$$

$$Q\left(L/hr\right)=5.57$$

$$V_{p,i}\left(L\right)=58.2\times \exp\left(\eta_{Vp,i}\right)$$

$$D_{1,i}\left(1/\text{hr}\right)=0.0722\times \exp\left({3.14\times \text{FED}+\eta }_{D1,i}\right)$$

$$ALAG_{1i}\left(\text{hr}\right)=0.189$$

$$F_{1,i}=1\times \exp\;\left(0.409\times \text{FED}\right)$$

When leritrelvir co-administrated with RTV, the pharmacokinetic parameters of leritrelvir are:

$${CL}_{i}\left(L/hr\right)=12.2\times \exp\left(-0.469\times \text{OC}1+0.412\times \text{OC}2-0.224\times \text{SEX}+OC1\times \eta_{OC1,i}+{OC2\times \eta }_{OC2,i}+\eta_{CL,i}\right)$$

$$V_{c,i}\left(L\right)=9.78\times \exp\left(OC1\times \eta_{OC1,i}+{OC2\times \eta }_{OC2,i}+\eta_{Vc,i}\right)$$

$$K_{a,i}\left(1/\text{hr}\right)=0.169\times \exp\left(0.291\times \text{FED}+\eta_{Ka,i}\right)$$

$$Q\left(L/hr\right)=0.611$$

$$V_{p,i}\left(L\right)=9.30\times \exp\left(\eta_{Vp,i}\right)$$

$$D_{1,i}\left(1/\text{hr}\right)=0.0684\times \exp\left({3.14\times \text{FED}+\eta }_{D1,i}\right)$$

$${ALAG}_{1,i}\left(\text{hr}\right)=0.166$$

$$F_{1,i}=1\times \exp\;\left(0.409\times \text{FED}\right)$$

**FIGURE 2:**
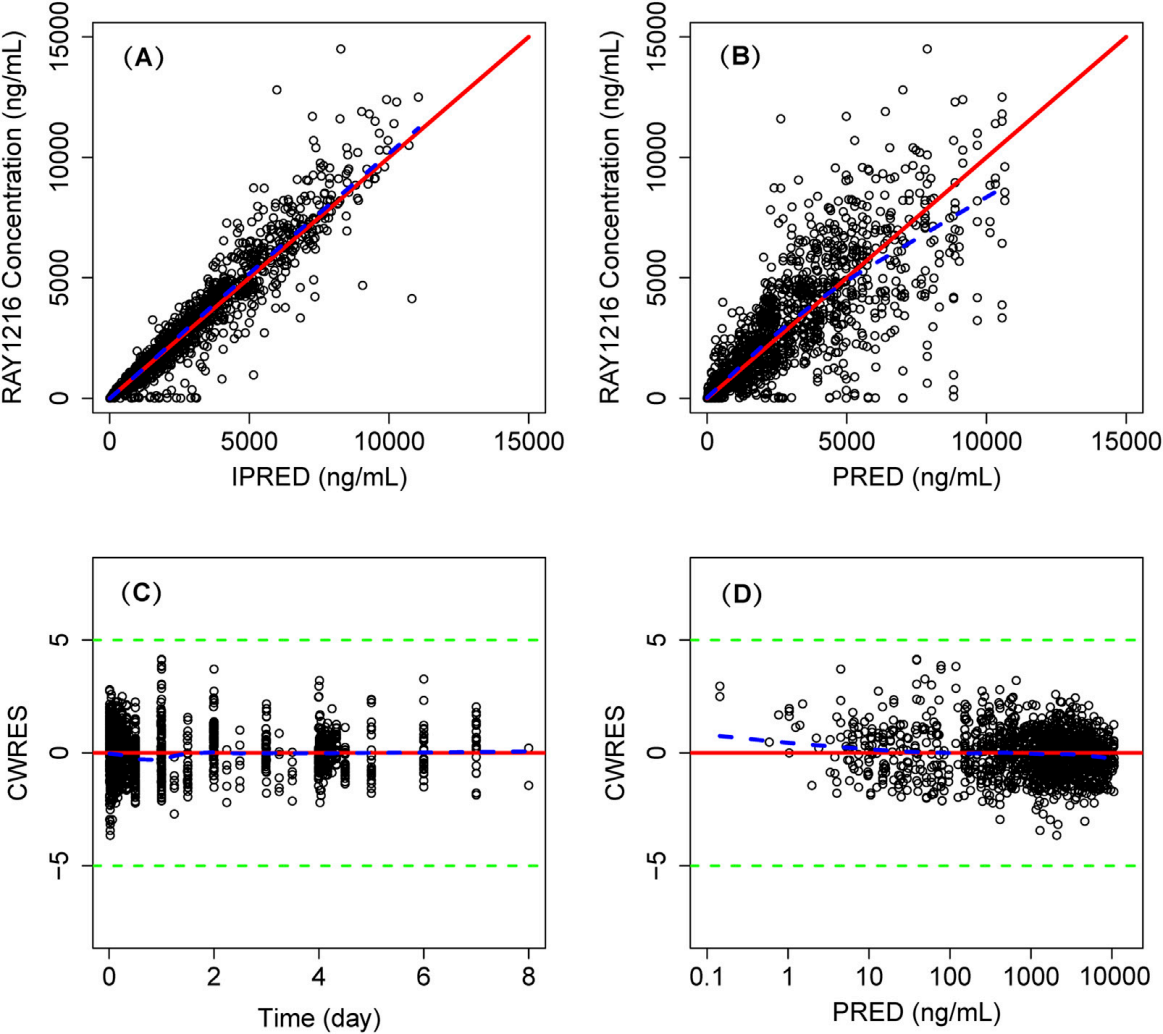
Goodness of fit plot of final population PK model. Individual predicted concentrations (IPRED) *versus* observed concentrations **(A)** and population predicted concentrations (PRED) *versus* observed concentrations **(B)**. Conditional weighted residuals (CWRES) *versus* time **(C)** and PRED **(D)**. Points are individual data. Red solid lines represent the unit diagonal (top) or line at zero (bottom). Blue dashed lines represent the lowess smooth curves. Green dashed lines represent the |CWRES| = 5.

**FIGURE 3:**
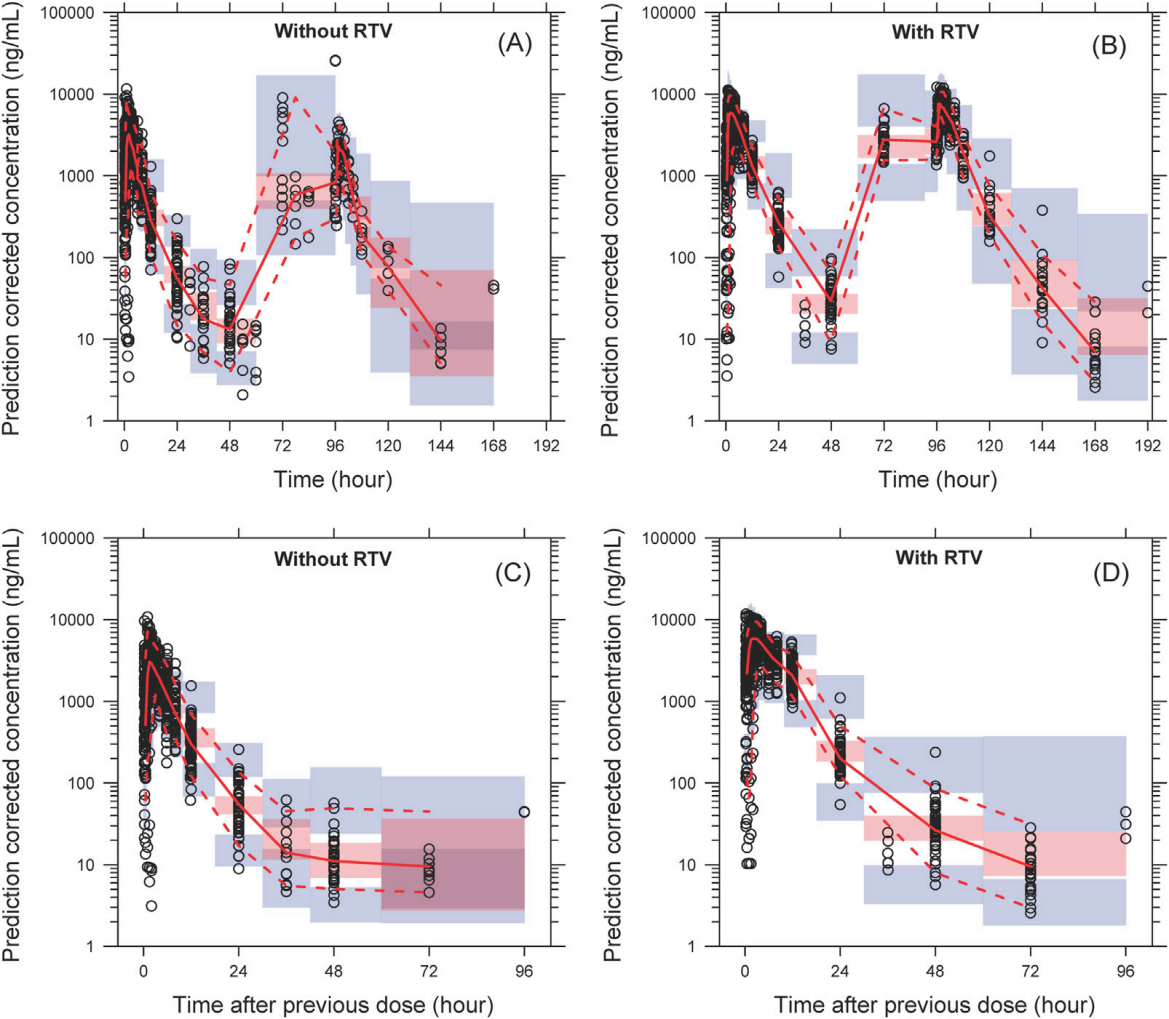
The pcVPC of plasma concentration–time profiles for leritrelvir alone **(A)** and co-administered with RTV **(B)** *versus* time. **(C, D)** are the pcVPC *versus* time after dose for leritrelvir alone and co-administered with RTV, respectively. Black circles are individual observed concentrations, solid red lines represent the median observed concentrations and dashed red lines represent the 2.5th and 97.5th percentiles of the observed concentrations over time. Pink-shaded areas represent the 95% confidence interval of the predicted median concentrations, and purple-shaded areas represent the 95% confidence interval of the predicted 2.5th and 97.5th percentiles of the concentrations over time.

The model successfully captured the observed plasma concentrations of leritrelvir in both single and co-administration with RTV scenarios. Goodness-of-fit plots demonstrated a strong agreement between predicted and observed leritrelvir concentrations, with residual plots indicating no apparent bias over time and predicted concentrations (Figure 2). pcVPC plots (Figure 3) that the median, 2.5th and 97.5th percentiles of observed values, lines, and dots fall within the 95% interval of the corresponding simulated median and 2.5th and 97.5th percentiles. This indicates that the final model aptly represents the central tendency and interindividual variability of plasma concentration distribution for leritrelvir under both conditions of single and co-administration with RTV. Bootstrap analysis results (see Table 2) show that the estimated values of the final model parameters highly overlap with the 95% confidence interval and the median and 95th percentile intervals of the bootstrap, further validating the robustness and stability of the final model.

### 3.3 PopPK model simulations

#### 3.3.1 Target concentration

**FIGURE 4:**
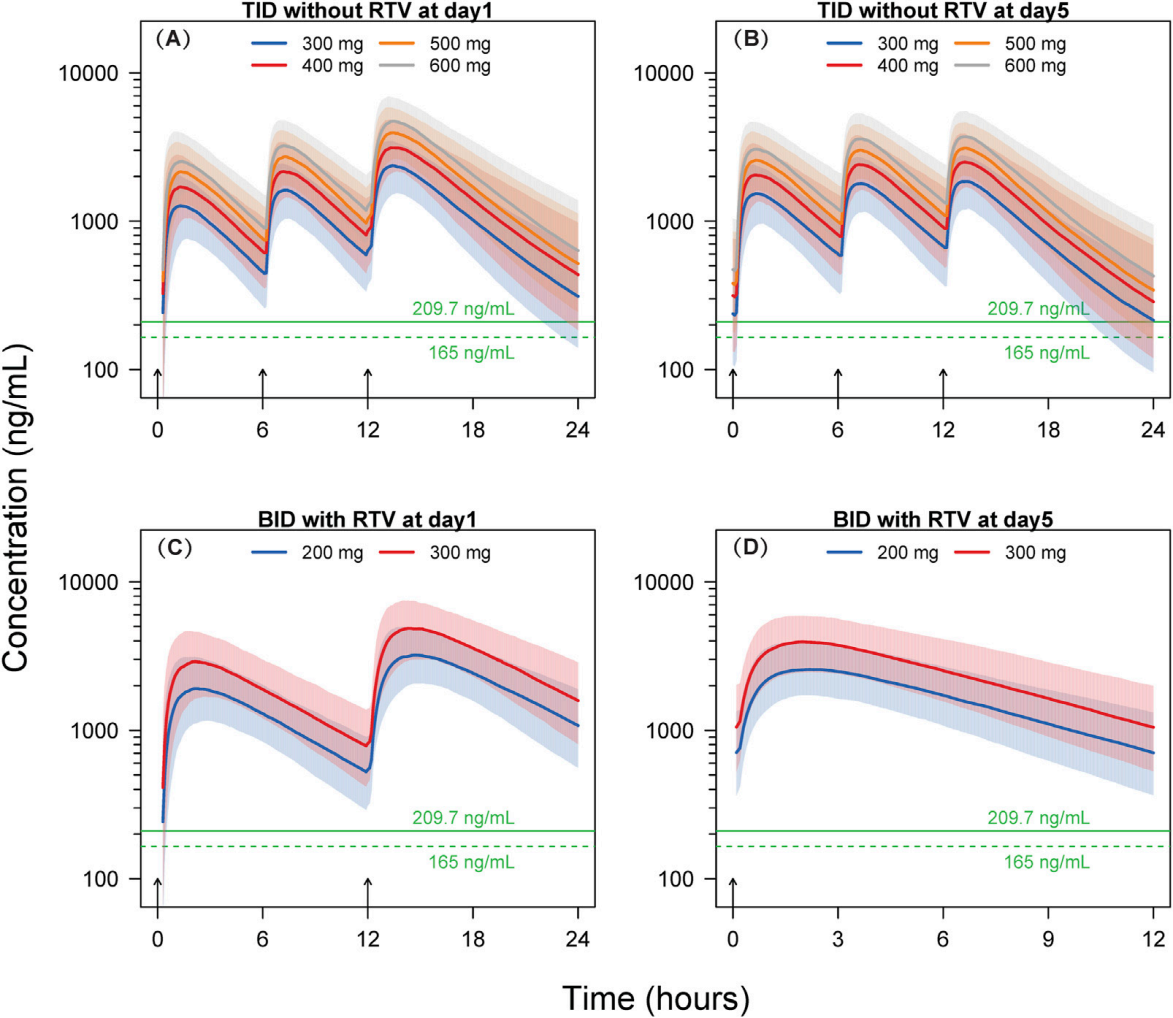
Simulated concentration-time profiles. Solid lines and shaded areas represent the median and 10^th^-90^th^ percentile interval of the simulated concentration-time profiles of individual subjects. The horizontal solid and dashed green lines represent the target concentrations of 209.7 ng/mL and 165 ng/mL, respectively. The short black arrows represent the dosing events. BID, twice-daily. TID, thrice-daily. RTV, co-administrated with 100 mg ritonavir. **(A)** TID without RTV at day 1; **(B)** TID without RTV at day 5; **(C)** BID with RTV at day 1; **(D)** BID with RTV at day 5.

The Results from the dosing regimen for Scenario 1 and Scenario 2 are shown in Supplementary Table S1 and Figure 4.

The findings of Scenario 1 indicate that, following the initial dose, a minimum of 97.8% of participants across the four dosage groups (300, 400, 500, and 600 mg) exhibit concentrations exceeding the EC_90_ threshold of 209.7 ng/mL at 6 h post-dosing. After the 15th dose on Day 5, at 12 h post-dosing, the proportions of subjects surpassing the EC_90_ in the 300, 400, 500, and 600 mg dosage groups are 51.5%, 69.9%, 80.8%, and 87.0%, respectively. Following the initial dose, at 6 h post-dosing, at an EC_90_ concentration of 165 ng/mL, a minimum of 99.4% of subjects within the four dosage groups (300, 400, 500, and 600 mg) exhibit concentrations exceeding the EC_90_. After the 15th dose on Day 5, at 12 h post-dosing, the percentages of subjects surpassing EC_90_ in the 300, 400, 500, and 600 mg dosage groups are 65.3%, 80.8%, 88.6%, and 94.5%, respectively.

In Scenario 2, leritrelvir is administered at doses of 200 and 300 mg BID at 8:00 and 20:00, respectively, for five consecutive days, in combination with RTV. Utilizing the simulated dosing regimen for Scenario 2, the results show that at EC_90_ concentrations of 209.7 ng/mL or 165 ng/mL, the proportions of subjects with trough concentrations exceeding EC_90_ in both dosage groups (200 and 300 mg) are close to 100% (≥98.6%).

#### 3.3.2 Impact of gender and food on exposure

The exposure of leritrelvir was calculated based on simulated concentration-time profiles for different genders and food consumption as depicted in Supplementary Figures S2, S3 and Supplementary Tables S2, S3. In comparison to male subjects, female subjects exhibited higher exposures ranging from 12.4% to 16.6% for leritrelvir alone and 32.8%–50.5% for leritrelvir co-administered with RTV. Furthermore, subjects in a fed state demonstrated increased exposures of 25.2%–63.0% for leritrelvir alone and 28.6%–33.7% for leritrelvir co-administered with RTV, in contrast to those in a fasted state.

## 4 Discussion

In this study, PK data from studies involving single and co-administered doses with RTV were adequately characterized using a two-compartment model with zero-order and first-order sequential absorption and first-order elimination. The drug concentration-time curve showed multiple peaks followed by a gradual decrease.

The study determined that leritrelvir demonstrated EC_90_ at a concentration of 209.7 ng/mL against the Omicron strain and the wild-type strain is 165 ng/mL. Utilizing these EC_90_ values and population pharmacokinetic simulation, dosing regimens were explored. The ratio of estimated clearance of leritrelvir to leritrelvir combined with RTV was 3.64 (44.4/12.2). It is evident that co-administration with RTV significantly enhances the exposure of leritrelvir by approximately 4-fold, indicating the need for a BID dosing schedule. In contrast, leritrelvir administered alone necessitates a TID dosing regimen.

The simulation results for thrice-daily dosing of leritrelvir suggest that a high percentage of participants (≥97.8%) exceeded the EC_90_ threshold after the first dose in all dosage groups at the 6-h mark, with EC_90_ concentrations of 209.7 ng/mL and 165 ng/mL. By the 5th day (15th dose) at 12 h post-dosing, the 300 mg group showed that 51.5% and 65.3% of subjects surpassed EC_90_ levels, while the 400 mg group had an increase to 69.9% and 80.8% exceeding EC_90_ level. Further dose escalation might increase these proportions; however, based on the simulated pharmacokinetic curves, it appears unnecessary. The trough concentrations at 6-h intervals for the initial two doses in the thrice-daily dosing regimen are notably higher than the corresponding EC_90_ values, as illustrated in Figure 4. Although the trough concentrations at 12-h intervals are lower, the duration below the EC_90_ threshold is shorter, indicating limited potential for further dose escalation. Therefore, a dosage of 400 mg three times daily could be appropriate for leritrelvir as a monotherapy. The findings from the simulation study on the co-administration of leritrelvir with ritonavir (RTV) twice daily indicate that for both dosage groups (200 and 300 mg), when the EC_90_ is either 209.7 ng/mL or 165 ng/mL, almost all subjects (≥98.6%) have trough concentrations exceeding the EC_90_ threshold. Consequently, the exposure achieved with a 200 mg dose of leritrelvir in combination with RTV is considered sufficient.

In order to further explore potential enhancements in the effectiveness of the 400 mg TID regimen, a Phase II trial was conducted involving COVID-19 patients (Wang et al., 2023). The trial consisted of three arms: leritrelvir administered alone at 400 mg TID, leritrelvir at 300 mg with 100 mg RTV given BID, and a Placebo group. Co-administration of 300 mg leritrelvir with RTV resulted in a trough concentration 5.6 times higher than the EC_90_ (209.7 ng/mL) in the current simulation, ensuring adequate exposure level.

The clinical trial results showed that patients treated with leritrelvir alone or in combination with RTV had shorter durations of viral shedding compared to the placebo group. Specifically, the duration was 166 h for leritrelvir alone and 155 h for leritrelvir with RTV, which were 4.2 days and 4.6 days shorter than the placebo group, respectively (Wang et al., 2023). These findings suggest that the efficacy of leritrelvir at a dosage of 400 mg three times a day is already optimized, and further improvements are unlikely. Given the potential risks of side effects of RTV, the leritrelvir standalone regimen at 400 mg TID remains the preferred treatment option.

In the pharmacokinetic analysis, gender and food consumption were identified as significant covariates. Gender affected CL while food consumption influenced F, K_a_, and D_1_. Females had higher exposure to leritrelvir than males, and subjects in a fed state had higher exposure compared to those in a fasted state for both leritrelvir alone and leritrelvir with RTV. Considering the wide safety margin, no dose adjustment of leritrelvir is necessary based on gender or food consumption.

In single-dose, multiple-dose, and food-effect studies, leritrelvir demonstrated a favorable safety and tolerability profile when administered as a monotherapy or in combination with ritonavir in healthy subjects. No dose-dependent adverse reactions were observed, with minimal incidence of adverse events in each treatment group, slightly higher than the placebo group. No Grade 3 or higher adverse events (AEs) were reported, no subjects prematurely withdrew from the study due to AEs, no serious adverse events (SAEs) occurred, and no meaningful changes were observed in subjects’ corrected QT (QTc) interval, pulse rate (PR) interval, heart rate, and blood pressure. These safety findings indicate that safety is not an important concern in the selection of a dosage regimen based on the current data.

The main limitations of this study include the fact that the subjects were healthy individuals, making it difficult to obtain efficacy data. Moreover, the duration of pharmacokinetic measurements was insufficient, given that the symptoms of mild to moderate COVID-19 patients generally resolve within a short period. Consequently, the study predominantly focused on a dosing duration of 5 days, which is inadequate for a thorough investigation of dynamic changes in auto-induction or auto-inhibition. This limitation is further compounded by the absence of data on interactions with other CYP3A4-metabolized drugs. Furthermore, the dataset excluded specific demographic groups, including the elderly and children, necessitating additional research on the pharmacokinetics within these populations. The existing pharmacokinetic model effectively characterizes the pharmacokinetic data from day 1 to day 5, as well as interindividual variability. Consequently, the outcomes of further analyses based on model simulations are deemed reliable.

According to the analysis above, the exposure level of leritrelvir 400 mg TID is expected to achieve good clinical efficacy, and the risk of drug interactions is significantly reduced when not co-administered with RTV. Therefore, a dosing regimen of 400 mg TID was employed in the Phase III trial of leritrelvir (Zhan et al., 2024). This trial was a randomized, double-blind, placebo-controlled, multicenter phase 3 trial conducted at 29 clinical sites in China during the early stages of the COVID-19 outbreak.

A total of 1359 patients with mild-to-moderate COVID-19 were enrolled, with 680 patients in the leritrelvir group and 679 patients in the placebo group (Zhan et al., 2024). The findings indicated that administering oral leritrelvir at a dose of 400 mg TID for 5 days led to a significant reduction in the time to sustained clinical recovery of all 11 COVID-19 symptoms by approximately 1 day (20.3 h) compared to the placebo (Zhan et al., 2024). Additionally, leritrelvir treatment resulted in a notable decrease in the SARS-CoV-2 viral load by approximately tenfold after a 3-day treatment period compared to the placebo (Zhan et al., 2024), which was consistent with findings from the nirmatrelvir/ritonavir trial (Hammond et al., 2022).

On 23 March 2023, the China National Medical Products Administration granted conditional approval for the marketing of leritrelvir tablets through a specialized drug approval pathway. This expedited approval, aside from meeting the urgent market demand, was also played a vital role in establishing an optimal dosage regimen through quantitative pharmacological auxiliary analysis. The insights gained from this analysis may serve as a valuable resource for subsequent research endeavors or clinical applications.

## 5 Conclusion

Leritrelvir pharmacokinetics was well characterized in healthy subjects. Clinical trial simulation analysis indicated that the trough concentration of leritrelvir 400 mg TID without RTV can ensure most of the subjects achieve the target level. Food consumption and gender were significant covariates, however, no dose adjustment is deemed necessary.

## Data Availability

The original contributions presented in the study are included in the article/Supplementary Material, further inquiries can be directed to the corresponding authors.
