# Supplementary material for: Model informed dose regimen optimizing in development of leritrelvir for the treatment of mild or moderate COVID-19
Source: Front Pharmacol. 2024 Aug 28;15:1449583. doi: 10.3389/fphar.2024.1449583 (PMC11387175; doi:10.3389/fphar.2024.1449583)
Supplement: Supplementary file 1 [file DataSheet1.docx]

Supplement

PopPK model development

Following a comparison of the 1-3 compartment models, the 2-compartment model was selected as the structural model for characterizing the PK of leritrelvir. Interindividual variability (IIV) in PK parameters was described using an exponential model:

| $\theta_{i}=e^{\left( \theta_{T}+\eta_{i} \right)}$ | (1) |
| --- | --- |

where *θ_i_* represents the parameter for the *i*^th^ subject, *θ_T_* denotes the natural logarithm of the typical value of the parameter within the population, and *η_i_* (ETA) is the random interindividual effect with mean 0 and variance ω^2^.

Residual error was described using proportional error model:

| ${C(t)}_{ij} = {Ĉ(t)}_{ij}\cdot(1+{}_{pij})$ | (2) |
| --- | --- |

Where *C(t)_ij_* and *Ĉ(t)_ij_* represent the *j^th^* observed concentration and the model-predicted concentration for the *i*^th^ subject, respectively; *ε_pij_* is the residual error with mean 0 and variance σ^2^.

The final PopPK model was determined based on a stepwise forward addition (p < 0.01) and backward deletion (p < 0.001) approach. Continuous covariates were normalized to the population median and modeled as following:

The impact of categorical covariates on pk parameter was modeled as following:

In the context of Pk modeling, the parameter *θ_i_* denotes the PK parameter of the ith subject, while *θ_T_* represents the natural logarithm of the population typical value of the PK parameter, *Cov_i_* is the continuous covariate value of the ith subject, *Cov_pop_* is the median of this continuous variable in the population. Additionally, Xi serves as the index for the categorical variable of the ith subject, where 0 value corresponds to the most prevalent category of the covariate, and other integer values correspond to alternative categories. *k_cov,I_* is the coefficient that describes the magnitude of the covariate's impact, ηi represents the random inter-individual variables subject to a normal distribution of mean 0 and variance ω^2^.

NONMEM Control Stream

$SUBROUTINE ADVAN4 TRANS4

$PK

IF (RTV.EQ.0) THEN

TVCL=THETA(1)

TVV2=THETA(3)

TVKA=THETA(5)

TVQ=THETA(7)

TVV3=THETA(9)

TVD1=THETA(11)

TVALAG1=THETA(13)

ELSE

TVCL=THETA(2)

TVV2=THETA(4)

TVKA=THETA(6)

TVQ=THETA(8)

TVV3=THETA(10)

TVD1=THETA(12)

TVALAG1=THETA(14)

ENDIF

OC1=0

OC2=0

IF (DOSESCH.GT.1.AND.TIME.GE.12 ) OC1=1 ; DOSESCH.GT.1 for multiple dose

IF (DOSESCH.GT.1.AND.TIME.GE.96) OC2=1

CL=EXP(TVCL+ETA(1)+OC1*THETA(18)+OC2*THETA(19)+OC1*ETA(8)+OC2*ETA(9)+THETA(20)*SEX)

V2=EXP(TVV2+ETA(2)+OC1*ETA(10)+OC2*ETA(11))

KA=EXP(TVKA+THETA(17)*FED+ETA(3))

Q=EXP(TVQ+ETA(4))

V3=EXP(TVV3+ETA(5))

D1=EXP(TVD1+THETA(16)*FED+ETA(6))

ALAG1=EXP(TVALAG1)

F1=EXP(THETA(15)*FED+ETA(7))

K=CL/V2

K23=Q/V2

K32=Q/V3

S2=V2/1000

$ERROR

IF(F.GT.0) THEN

IPRED=F

ELSE

IPRED=0.005

ENDIF

IF (TFDS.LE.2.AND.FED.EQ.1) THEN ;TFDS: time after previous dose

Y=IPRED*(1+EPS(2))

ELSE

Y=IPRED*(1+EPS(1))

ENDIF

**Supplementary Table**

**Table S1.** Summary of simulated results of leritrelvir.

| **Group** | **Number of dose** | **TAD (hr)** | **C_trough_ (ng/mL)** | **N% of C_trough_ above EC90** | | | | | |
| --- | --- | --- | --- | --- | --- | --- | --- | --- | --- |
|  |  |  |  | **EC_90_=209.7 ng/mL** | | | **EC_90_=165 ng/mL** | | |
|  |  |  | **Median (10^th^-90^th^ percentile)** | **>EC_90_** | **>3*EC_90_** | **>5*EC_90_** | **>EC_90_** | **3*EC_90_** | **>5*EC_90_** |
| 300 mg TID | 1^st^ | 6 | 455 (269-746) | 97.8 | 20.3 | 2.1 | 99.4 | 41.7 | 6.6 |
|  | 2^nd^ | 6 | 633 (373-1019) | 99.5 | 50.4 | 9.2 | 100.0 | 72.6 | 24.8 |
|  | 3^rd^ | 12 | 311 (140-650) | 74.7 | 10.9 | 1.9 | 85.4 | 23.0 | 5.1 |
|  | 13^th^ | 6 | 600 (332-1017) | 99.1 | 45.1 | 9.2 | 99.9 | 66.1 | 22.2 |
|  | 14^th^ | 6 | 677 (375-1148) | 99.4 | 55.6 | 15.1 | 99.9 | 75.0 | 32.5 |
|  | 15^th^ | 12 | 215 (96-444) | 51.5 | 3.7 | 0.3 | 65.3 | 7.2 | 0.8 |
| 400 mg TID | 1^st^ | 6 | 624 (362-1065) | 98.8 | 49.6 | 10.9 | 99.7 | 71.4 | 24.8 |
|  | 2^nd^ | 6 | 860 (491-1447) | 99.9 | 76.6 | 31.1 | 100.0 | 89.6 | 53.2 |
|  | 3^rd^ | 12 | 436 (184-976) | 86.7 | 27.1 | 8.3 | 92.2 | 42.4 | 15.4 |
|  | 13^th^ | 6 | 809 (448-1508) | 99.6 | 72.2 | 29.7 | 100.0 | 85.1 | 48.1 |
|  | 14^th^ | 6 | 910 (501-1711) | 99.8 | 79.3 | 38.1 | 100.0 | 90.1 | 58.9 |
|  | 15^th^ | 12 | 287 (119-686) | 69.9 | 12.0 | 1.8 | 80.8 | 20.5 | 5.4 |
| 500 mg TID | 1^st^ | 6 | 762 (456-1276) | 99.9 | 68.9 | 21.2 | 100.0 | 85.6 | 41.5 |
|  | 2^nd^ | 6 | 1035 (626-1728) | 100.0 | 89.5 | 48.9 | 100.0 | 96.7 | 71.9 |
|  | 3^rd^ | 12 | 517 (245-1124) | 93.9 | 37.2 | 12.4 | 97.3 | 53.0 | 22.3 |
|  | 13^th^ | 6 | 990 (562-1750) | 100.0 | 85.6 | 43.0 | 100.0 | 94.7 | 66.1 |
|  | 14^th^ | 6 | 1115 (633-1985) | 100.0 | 90.2 | 56.4 | 100.0 | 96.9 | 75.9 |
|  | 15^th^ | 12 | 343 (159-761) | 80.8 | 17.4 | 3.9 | 88.6 | 28.4 | 8.0 |
| 600 mg TID | 1^st^ | 6 | 920 (569-1528) | 100.0 | 84.1 | 37.6 | 100.0 | 94.1 | 59.5 |
|  | 2^nd^ | 6 | 1260 (771-2106) | 100.0 | 95.8 | 66.9 | 100.0 | 98.8 | 86.6 |
|  | 3^rd^ | 12 | 633 (297-1379) | 96.4 | 50.7 | 21.2 | 98.4 | 65.8 | 34.1 |
|  | 13^th^ | 6 | 1206 (713-2138) | 100.0 | 93.5 | 61.1 | 100.0 | 98.0 | 81.5 |
|  | 14^th^ | 6 | 1360 (790-2449) | 100.0 | 95.6 | 71.3 | 100.0 | 98.7 | 86.9 |
|  | 15^th^ | 12 | 426 (192-942) | 87.0 | 27.5 | 7.7 | 94.5 | 41.3 | 14.7 |
| 300 mg BID + RTV | 1^st^ | 12 | 817 (446-1395) | 99.5 | 71.6 | 28.8 | 99.9 | 86.2 | 48.9 |
|  | 2^nd^ | 12 | 1585 (807-2862) | 100.0 | 96.0 | 78.7 | 100.0 | 98.7 | 88.9 |
|  | 9^th^ | 12 | 1047 (530-2000) | 99.8 | 83.9 | 49.8 | 100.0 | 92.3 | 68.6 |
| 200 mg BID + RTV | 1^st^ | 12 | 543 (312-911) | 98.6 | 36.1 | 5.7 | 99.5 | 58.0 | 15.9 |
|  | 2^nd^ | 12 | 1077 (561-1889) | 100.0 | 85.8 | 52.3 | 100.0 | 93.1 | 71.1 |
|  | 9^th^ | 12 | 705 (366-1320) | 99.3 | 59.0 | 21.6 | 99.8 | 77.3 | 37.5 |

RTV, co-administrated with 100 mg ritonavir. BID, twice-daily. TID, thrice-daily.

**Table S2.**

|  | | **400 mg TID** | | **300 mg + 100 mg RTV BID** | |
| --- | --- | --- | --- | --- | --- |
|  |  | Fasted (n=42) | Fed (n=12) | Fasted (n=36) | Fed (n=12) |
| AUC_1_ (ng*day/mL) | Geometric Mean (% CV) | 1417 (31.1) | 2297 (23.2) | 2739 (28.3) | 3584 (26) |
|  | Difference (%)^a^ | — | 62.1 | — | 30.8 |
| C_max1_ (ng/mL) | Geometric Mean (% CV) | 3255 (29.9) | 5307 (17.7) | 5597 (31.8) | 7304 (25.7) |
|  | Difference (%)^a^ | — | 63.0 | — | 30.5 |
| C_min1_ (ng/mL) | Geometric Mean (% CV) | 413 (47.6) | 542 (62.9) | 1639 (37.1) | 2191 (35.5) |
|  | Difference (%)^a^ | — | 31.4 | — | 33.7 |
| AUC_ss_  (ng*day/mL) | Geometric Mean (% CV) | 1321 (32.2) | 2126 (25.2) | 2592 (30.7) | 3334 (26.7) |
|  | Difference (%)^a^ | — | 60.9 | — | 28.6 |
| C_max,ss_  (ng/mL) | Geometric Mean (% CV) | 2565 (30.8) | 4042 (20.5) | 4366 (32.4) | 5664 (26.8) |
|  | Difference (%)^a^ | — | 57.6 | — | 29.7 |
| C_min,ss_  (ng/mL) | Geometric Mean (% CV) | 275 (48.6) | 345 (59.3) | 1047 (42.9) | 1356 (34.2) |
|  | Difference (%)^a^ | — | 25.2 | — | 29.5 |
| Gender | Male (%) | 21 (50) | 6 (50) | 18 (50) | 6 (50) |
|  | Female (%) | 21 (50) | 6 (50) | 18 (50) | 6 (50) |
| ^a^difference from the geometric mean of simulated exposures of subjects in fasted state. BID, twice-daily. TID, thrice-daily. | | | | | |

**Table S3.**

|  | | **400 mg TID** | | **300 mg + 100 mg RTV BID** | |
| --- | --- | --- | --- | --- | --- |
|  |  | Male (n=27) | Female (n=27) | Male (n=24) | Female (n=24) |
| AUC_1_ (ng*day/mL) | Geometric Mean (% CV) | 1465 (35.2) | 1700 (35.1) | 2542 (30.5) | 3376 (24.3) |
|  | Difference (%)^a^ | — | 16.0 | — | 32.8 |
| C_max1_ (ng/mL) | Geometric Mean (% CV) | 3359 (33) | 3919 (33.1) | 5159 (32.8) | 6937 (26) |
|  | Difference (%)^a^ | — | 16.6 | — | 34.5 |
| C_min1_ (ng/mL) | Geometric Mean (% CV) | 414 (60.4) | 465 (52) | 1459 (31.7) | 2128 (34.2) |
|  | Difference (%)^a^ | — | 12.4 | — | 45.8 |
| AUC_ss_  (ng*day/mL) | Geometric Mean (% CV) | 1370 (36) | 1574 (36.4) | 2356 (30.9) | 3235 (25.1) |
|  | Difference (%)^a^ | — | 14.9 | — | 37.3 |
| C_max,ss_  (ng/mL) | Geometric Mean (% CV) | 2634 (33.5) | 3058 (33.2) | 4017 (32.5) | 5405 (27.1) |
|  | Difference (%)^a^ | — | 16.1 | — | 34.5 |
| C_min,ss_  (ng/mL) | Geometric Mean (% CV) | 271 (56.9) | 309 (51) | 911 (33.4) | 1370 (36.2) |
|  | Difference (%)^a^ | — | 13.8 | — | 50.5 |
| Food consumpsion | Fasted (%) | 21 (77.8) | 21 (77.8) | 18 (75) | 18 (75) |
|  | Fed (%) | 6 (22.2) | 6 (22.2) | 6 (25) | 6 (25) |

^a^difference from the geometric mean of simulated exposures of male subjects. BID, twice-daily. TID, thrice-daily.

**Supplementary Figure**

**Figure S1.**


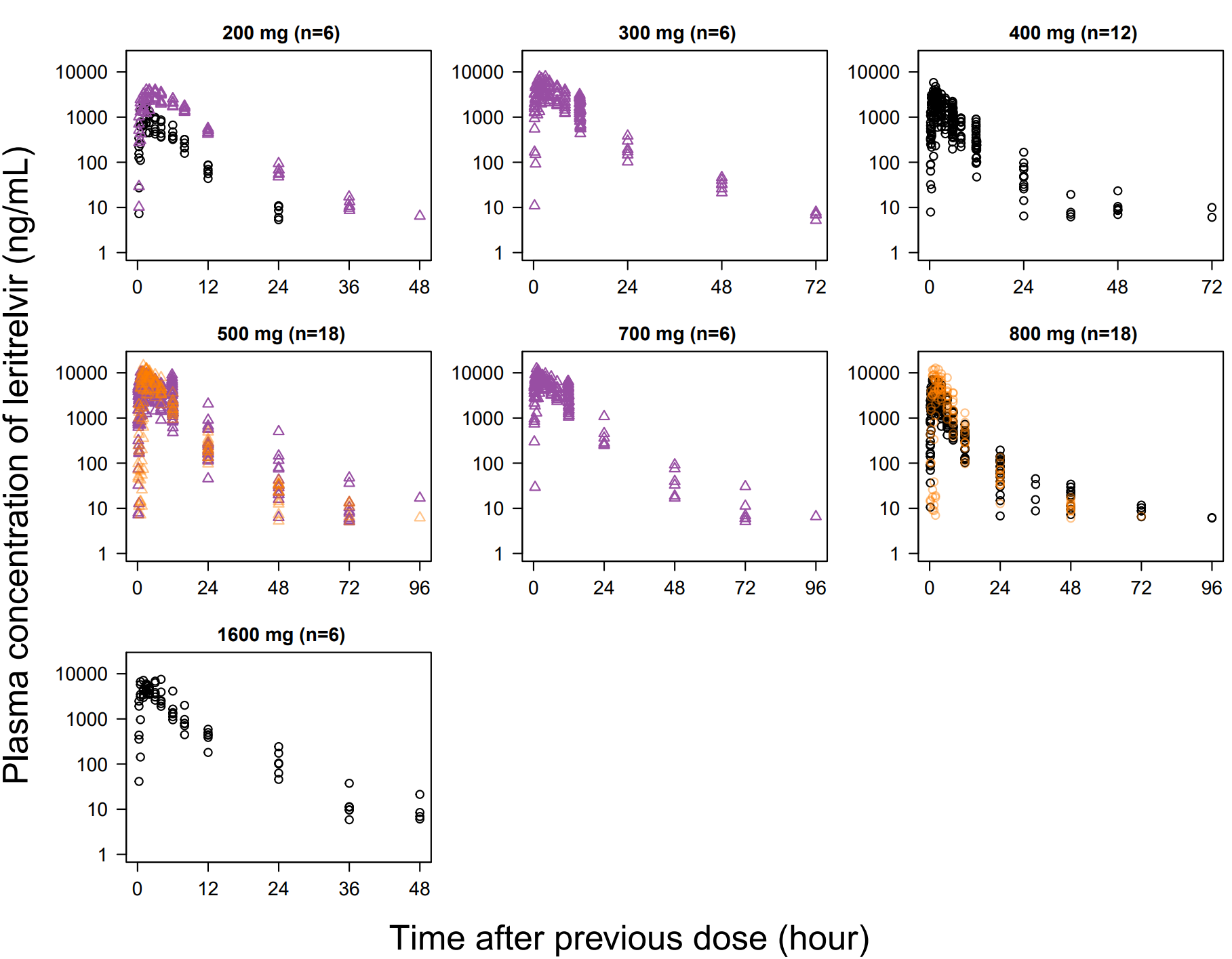


Concentration-time profile stratified by dose arm. The black and orange circles represent leritrelvir alone in fasted and fed condition. The purple and orange triangles represent leritrelvir co-administrated with ritonavir.

**Figure S2.**


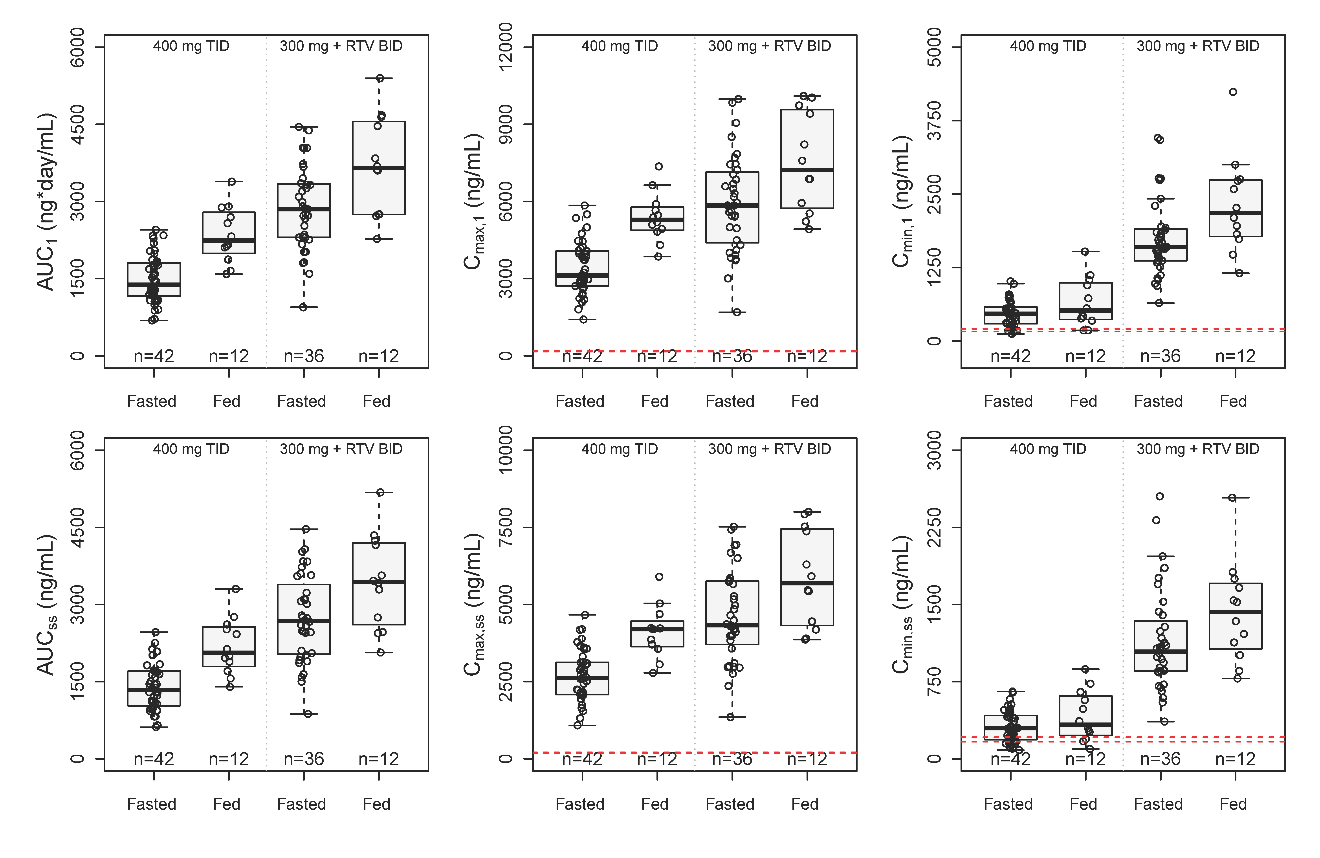


The impact of food on exposures. The circles represent the pharmacokinetic exposure simulated by the model. The horizontal line in the middle of the box plot is the median. The lower and upper edges of the box are the 25th and 75th percentiles respectively. The upper and lower horizontal lines are the distances from the edge of the box. The maximum and minimum values of data exceeding 1.5 times the IQR (upper and lower interquartile range), the red dashed lines are two EC90 values, 209.7 ng/mL (higher) and 165 ng/mL (lower), respectively.

**Figure S3.**


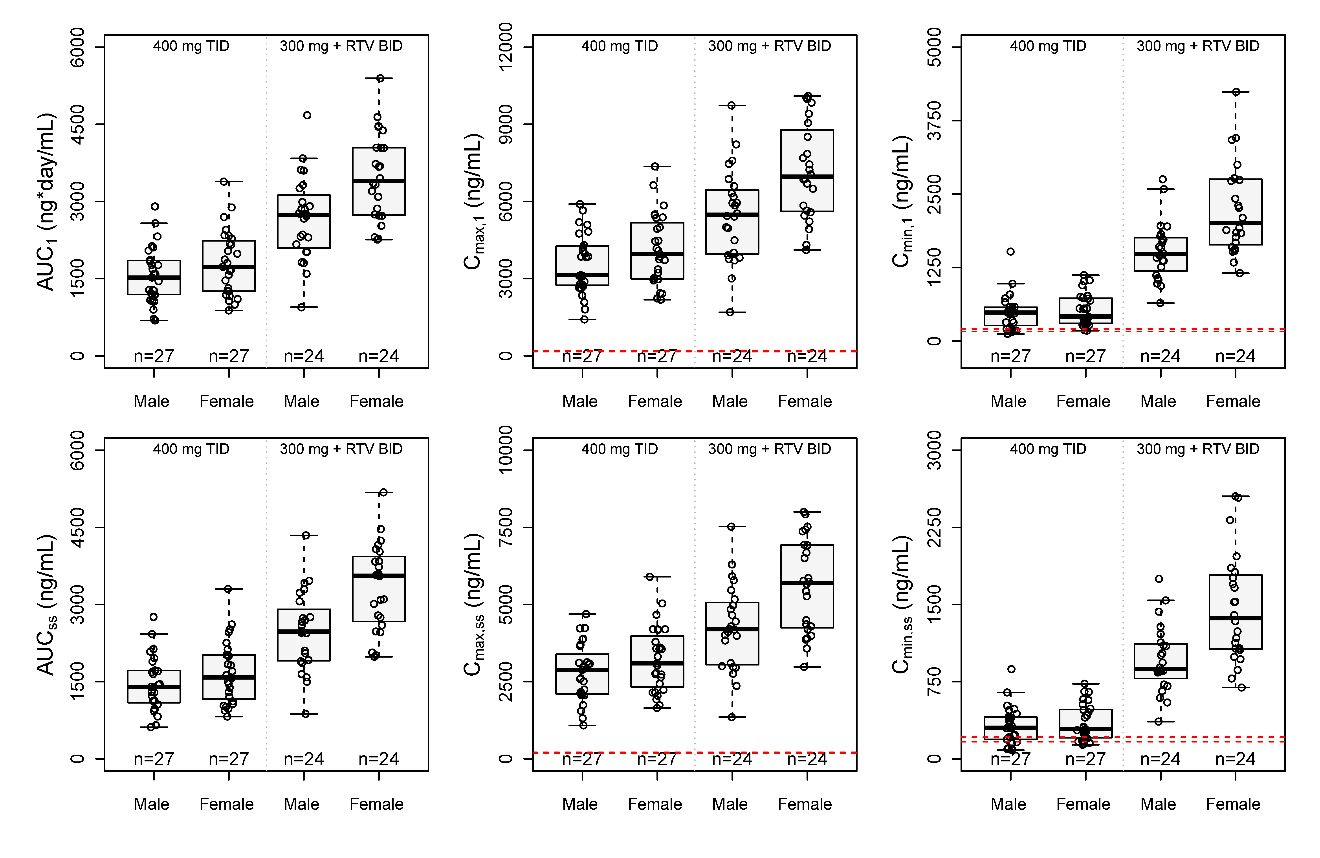


The impact of gender on exposures. The circles represent the pharmacokinetic exposure simulated by the model. The horizontal line in the middle of the box plot is the median. The lower and upper edges of the box are the 25th and 75th percentiles respectively. The upper and lower horizontal lines are the distances from the edge of the box. The maximum and minimum values of data exceeding 1.5 times the IQR (upper and lower interquartile range), the red dashed lines are two EC90 values, 209.7 ng/mL (higher) and 165 ng/mL (lower), respectively. BID, twice-daily. TID, thrice-daily.
